# Knowledge graph and emerging trends in oxidative stress research on hepatic ischemia-reperfusion injury: a bibliometric analysis (1995–2024)

**DOI:** 10.3389/fphar.2025.1587591

**Published:** 2025-06-18

**Authors:** Simeng Lei, Yangkai Fu, Bo Zhang, Hanwen Yang, Zhili Ji

**Affiliations:** Department of Hepatobiliary and Pancreaticosplenic Surgery, Beijing Chaoyang Hospital, Capital Medical University, Beijing, China

**Keywords:** hepatic ischemia reperfusion injury, bibliometric analysis, global trends, research hotspot, oxidative stress

## Abstract

**Background:**

Hepatic ischemia-reperfusion injury (HIRI) is a common complication in surgical procedures such as liver transplantation and extensive hepatectomy, characterized by an ischemic or hypoxic phase followed by reperfusion. Oxidative stress, primarily resulting from an imbalance between the generation and clearance of reactive oxygen species (ROS), plays a pivotal role in HIRI pathogenesis and has garnered significant research attention.

**Objective:**

This bibliometric analysis comprehensively reviews global research trends and priorities in the study of oxidative stress in HIRI from 1995 to 2024, providing valuable insights and guidance for future researchers.

**Methods:**

We conducted a systematic bibliometric analysis of relevant publications indexed in the Web of Science Core Collection (1995-2024), employing specific search qualifiers. Analyses were performed using CiteSpace (version 6.2.R6) and VOSviewer (version 1.6.20).

**Results:**

Final analysis included 2,367 publications. Over the past three decades, annual publication numbers in this field have consistently risen. China, the United States, and Turkey emerged as the leading contributing countries. Wuhan University, Sun Yat-sen University, and Shanghai Jiao Tong University ranked as the top three institutions by publication volume. The *Journal of Surgical Research* published the most articles, followed by *Transplantation Proceedings* and *Free Radical Biology and Medicine*. Rosello Catafau Joan, Lee Sun Mee, and Ye Qifa were identified as the most prolific authors. High-frequency keywords included “oxidative stress”, “ischemia reperfusion injury”, and “liver”.

**Conclusion:**

Our findings indicate a shift in research focus from elucidating fundamental mechanisms towards exploring therapeutic interventions and associated protective effects. Nanotechnology and epigenetic modifications represent promising future avenues for treating HIRI in the therapeutic domain.

## 1 Introduction

Liver disease is the focus of dedicated research by healthcare professionals around the world and is responsible for more than 2 million deaths annually, or 4% of global deaths (Devarbhavi et al., 2023). Liver transplantation is now an established treatment for acute, chronic, metabolic and end-stage liver disease such as hepatocellular carcinoma, and partial hepatectomy remains one of the few treatment options for patients with oncological liver disease (Brandel et al., 2022; Sugawara and Hibi, 2024). Both surgical procedures have a common complication: Hepatic ischemia-reperfusion injury (HIRI) (Jin et al., 2023). HIRI is a reperfusion injury caused by inadequate or complete interruption of blood supply and subsequent restoration of blood supply, a process that leads to hepatocyte damage, apoptosis and dysfunction. The pathomechanisms of HIRI are highly complex and not fully understood. Potential mechanisms currently proposed include mitochondrial damage, oxidative stress imbalance, aberrant cell death, immune cell hyperactivation, intracellular inflammation, and other complex events (Li R. et al., 2024; Liu J. et al., 2024). Oxidative stress has been shown to be a key factor in the mechanisms underlying HIRI (Tong et al., 2023; de Oliveira and Gonçalves, 2025; Yu et al., 2025). The study of oxidative stress in HIRI has become an important area of research in liver medicine.

Oxidative stress refers to the imbalance between reactive oxygen species (ROS) and antioxidants in the body, which leads to lipid peroxidation of cell membranes, resulting in cell damage and ultimately cell death (Su et al., 2019; Juan et al., 2021).

During HIRI, ROS are generated from many different sources, including the mitochondrial electron transport chain, xanthine oxidase, NADPH oxidase and uncoupled nitric oxide synthase (NOS) (Uehara et al., 2005). Studies have shown that oxidative stress not only directly damages cellular structure and function, but also exacerbates liver injury through a variety of mechanisms, such as regulating cell signaling pathways, promoting inflammatory responses, and influencing apoptosis, and that ROS also affect the expression of downstream genes or proteins and influence the process of HIRI (Chang et al., 2017; Tang et al., 2022; Liu J. et al., 2024) Since oxidative stress plays an important role in HIRI, intervention strategies targeting oxidative stress have become an important therapeutic approach to improve the prognosis of HIRI. Currently reported therapeutic approaches targeting oxidative stress include Inhibition of γ-glutamyl transpeptidase (Tamura et al., 2016; Kubota et al., 2020), Melatonin (Kireev et al., 2013; Mao et al., 2022), Trimetazidine (Elimadi et al., 1998), Glutathione (Schauer et al., 2004), Epigallocatechin-3-gallate (Tak et al., 2016), Resveratrol (Wang et al., 2023), Genistein (Akinci et al., 2019). Many of these strategies are currently at the experimental animal model stage. More systematic and comprehensive studies are needed to elucidate their mechanisms, clinical significance, and efficacy. However, it cannot be denied that the relationship between HIRI and oxidative stress has received more and more attention with the in-depth study of HIRI.

To tackle this challenge, we conducted a comprehensive bibliometric analysis of oxidative stress research in HIRI. This approach allows for a quantitative assessment of publication trends, the identification of key research clusters, and the visualization of interdisciplinary linkages that may be overlooked in traditional narrative reviews. This study not only provides a comprehensive view of the literature for researchers concerned with the effects of oxidative stress in HIRI, helping them to grasp the research trends and future directions, but also provides strong theoretical support for clinical practice and promotes the development of more effective treatment strategies. By exploring this field in depth, we hope to make a positive contribution to improving the prognosis and quality of life of patients with HIRI.

## 2 Methods

### 2.1 Data collection and search strategy

**FIGURE 1:**
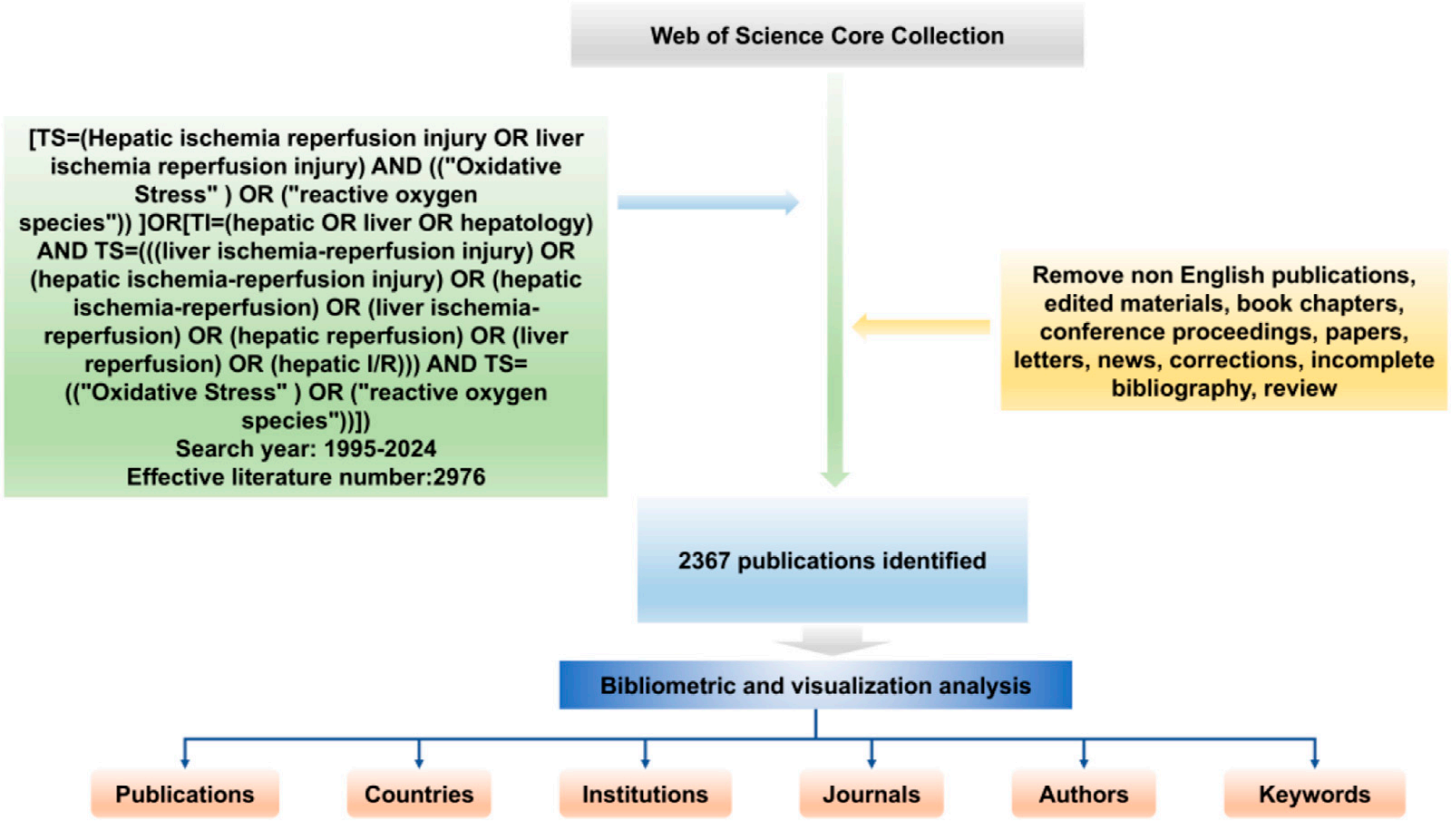
Flowchart for including and excluding publications.

The Web of science database is a comprehensive information service platform that facilitates interdisciplinary literature searches, and the database is widely recognized as providing many indexed publications that are often used for bibliometric analysis. In this paper, the Web of Science Core Collection (WoSCC) was selected as the literature source. The period searched was 1995–2024. To ensure the accuracy of the literature and topics included in the analysis, a title search was used with the following strategy: [TS=(Hepatic ischemia reperfusion injury OR liver ischemia reperfusion injury) AND ((“Oxidative Stress”) OR (“reactive oxygen species”)) ] OR [TI=(hepatic OR liver OR hepatology) AND TS=(((liver ischemia-reperfusion injury) OR (hepatic ischemia-reperfusion injury) OR (hepatic ischemia-reperfusion) OR (liver ischemia-reperfusion) OR (hepatic reperfusion) OR (liver reperfusion) OR (hepatic I/R))) AND TS= ((“Oxidative Stress”) OR (“reactive oxygen species”))]. A total of 2976 studies were retrieved and then the literature titles were manually screened according to the established inclusion and exclusion criteria, which included incomplete information about authors and institutions, ambiguous year of publication, as well as incomplete keywords and duplicate publications, and excluded literature unrelated to the topic, such as conference papers, news reports, and newsletters. Only 2367 articles exported records and cited references in plain text file format. All data used in this work were downloaded from public databases and therefore did not require ethics committee approval or informed consent. The detailed search strategy and analysis content are shown in Figure 1.

### 2.2 Statistical analysis

Information including annual publications and journal distribution was obtained from the WoSCC database, and the annual publications graph was generated using Origin Pro 2024. We chose VOSviewer 1.6.20 software to batch import the English literature data and dynamically adjust the author’s publication value to achieve the best display effect. We selected Overlay Visualization and Network Visualization for visualization and analysis. When setting other software parameters, we selected Association Strength as the parameter in the Method functional area and dynamically adjusted the Attraction and Repulsion parameters to achieve the best display effect. At the same time, we dynamically adjusted the Scale, Labels, Lines and Colors parameters in the Visualization Display functional area to make the plots clearer and nicer. In the bibliometric analysis of the dataset using the bibliometric visualization software CiteSpace 6.2R6, we selected the “Full Record with Cited References” option. We then exported the fully documented and cited reference data from the WoSCC database to a plain text file and imported it into CiteSpace. In terms of parameterization, we divided the time slices by 1 year and selected the cosine algorithm to compute the uniform strength of the network and extract the top 10% of targets in each time slice. We selected the node type and set the keyword threshold (Top N) to 50, the k metric (k = 25) was used for the extraction node threshold, and default values were used for all other parameters. Pathfinder and pruned slice networks were used to crop each slice and merge the networks to highlight important network structures. To cluster the literature keywords, the LSI algorithm was used to identify the top-ranked title words as cluster labels.

## 3 Results

### 3.1 Annual analysis of article count and trends

**FIGURE 2:**
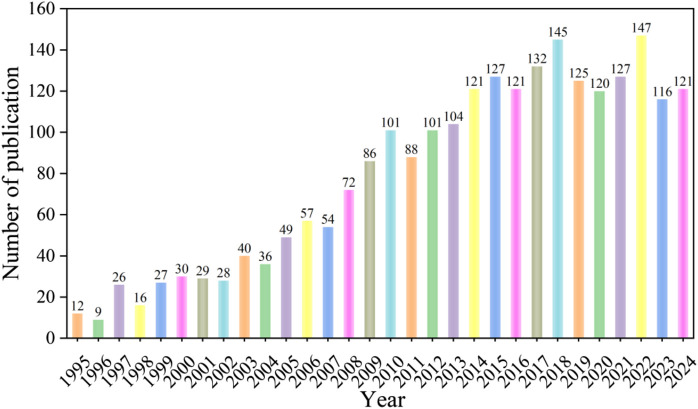
Number and trend of publications on oxidative stress in hepatic ischemia-reperfusion injury published between 1995 and 2024.

Figure 2 shows the bibliometric analysis of publication data on oxidative stress in HIRI from 1995 to 2024. The annual number of articles has generally shown an upward trend over the past 30 years, with a significant increase in 2008 and 2022 compared to the previous year. Notably, from 2005 to 2014, the number of articles grew at the fastest rate in the decade. Since 2014, the annual number of articles has shown a stable trend. The highest annual number of articles in the last 30 years occurred in 2022.

### 3.2 Analysis of cooperation interactions between key countries and institutions

**FIGURE 3:**
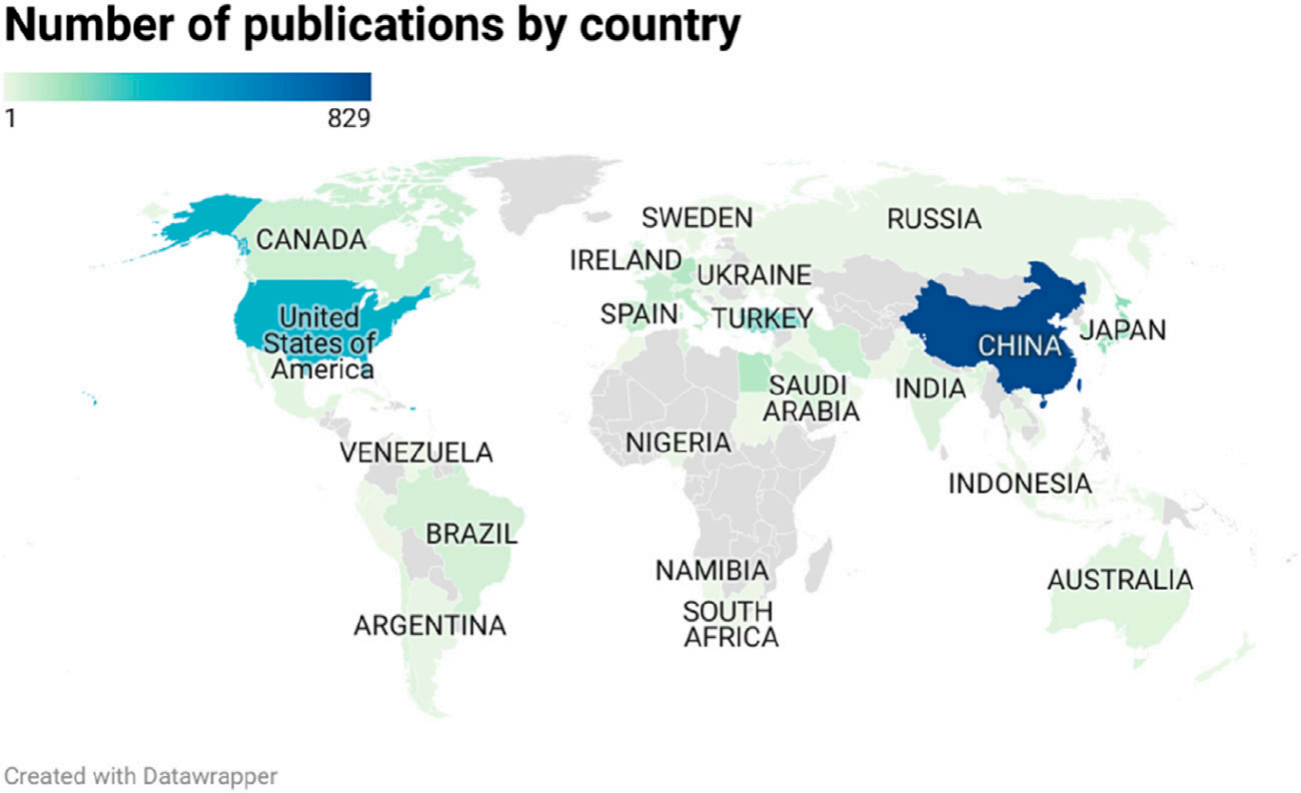
World map of the range of the main research countries, Dark blue represents a high volume of items, light green represents a low volume of items.

**FIGURE 4:**
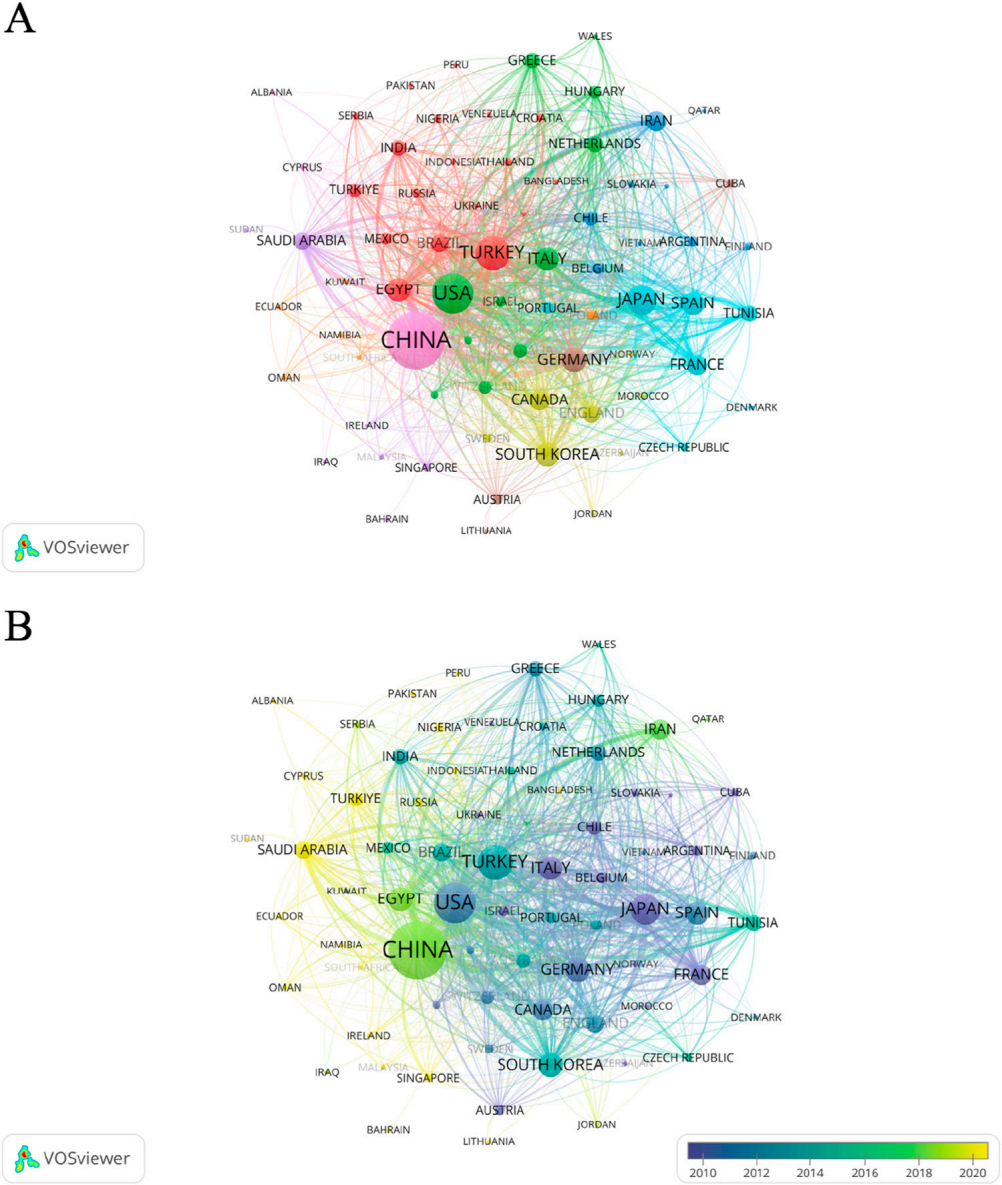
Collaborative network mapping study between countries/regions from 1995 to 2024. **(A)** Collaboration between countries/regions on the topic of oxidative stress in hepatic ischemia-reperfusion injury, data exported from Vosviewer. **(B)** Network diagram of collaborations involving countries in the study of oxidative stress in hepatic ischemia-reperfusion injury. The size of the circle indicates the number of articles published in each country, and the width of the connecting lines indicates the degree of collaboration between countries. Yellow color indicates that the articles were published recently, while purple nodes represent earlier publications.

We statistically analyzed the number of articles on oxidative stress in HIRI in major countries around the world from 1995 to 2024. The results showed that China, the United States, and Turkey were the top three countries in terms of the number of articles, publishing 829, 374, and 243 articles, respectively (Figure 3) Meanwhile, we noticed that the citation statistics show that the top three countries are the United States, China and Japan. The number of publications and the number of citations is not the same (Supplementary Table S1). In addition, Figure 4 shows a network relationship diagram based on the cooperation between countries. China and the United States, as the core countries, dominate the global research cooperation. The size of the nodes reflects the frequency and influence of their cooperation, and the thicker the connection, the higher the intensity of cooperation. Figure 4A shows the clustering of collaboration. The groups represented by different colors reflect the regional cooperation characteristics, the regional cooperation relationship between the United States and Italy, the Netherlands and Germany, and the regional cooperation relationship between Turkey and Egypt and Brazil. The time view of Figure 4B shows that over the past 30 years, traditional scientific research powers such as Japan, Chile, Germany, Italy, and the United States have been more active in early cooperation, while emerging economies such as China, Saudi Arabia, Egypt, and Turkey have shown a significant growth trend in recent years.

**FIGURE 5:**
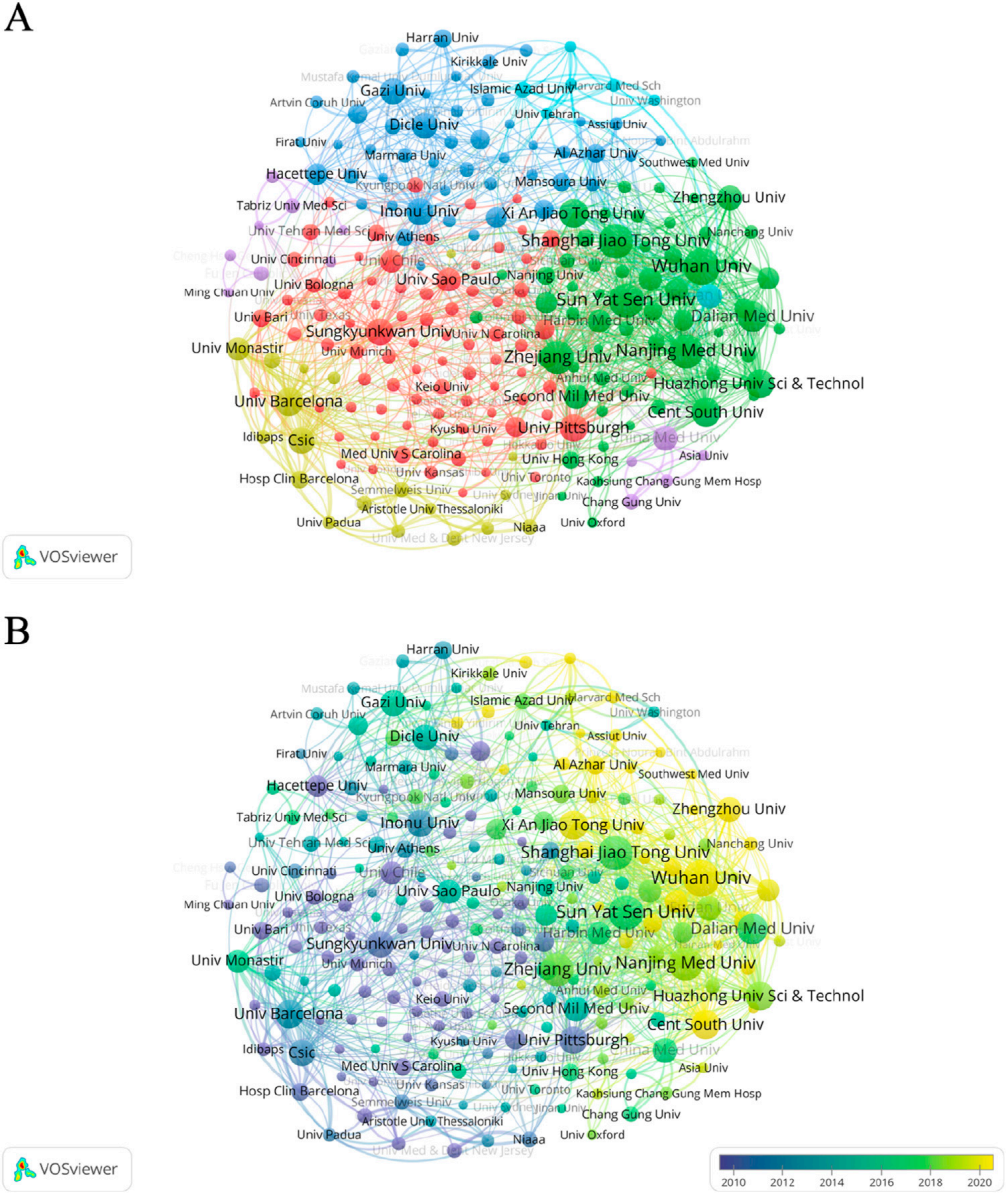
Network map of collaborations between institutions from 1995 to 2024. **(A)** Collaboration between institutions on the topic of oxidative stress in hepatic ischemia-reperfusion injury. **(B)** Collaborative network diagram of institutions involved in the study of oxidative stress in hepatic ischemia-reperfusion injury. The size of the circle indicates the number of articles published by each institution, and the width of the connecting lines indicates the degree of collaboration between institutions. Yellow color indicates that institutions published these articles recently, while purple nodes represent institutions that published these articles earlier.

In the past 30 years, Wuhan University, Sun Yat Sen University, and Shanghai Jiaotong University ranked first, second, and third in terms of publication volume, with 45, 42, and 41 articles, respectively. The publication time of the three universities was mostly in the last decade. However, we can find that from the citation statistics, the top three institutions are University of Pittsburgh, Shanghai Jiao Tong University and Dalian Medical University. Among these schools, only Shanghai Jiao Tong University ranks among the top three in terms of number of publications and citations. (Figure 5; Supplementary Table S2).

### 3.3 Analysis of disciplines and journals

**FIGURE 6:**
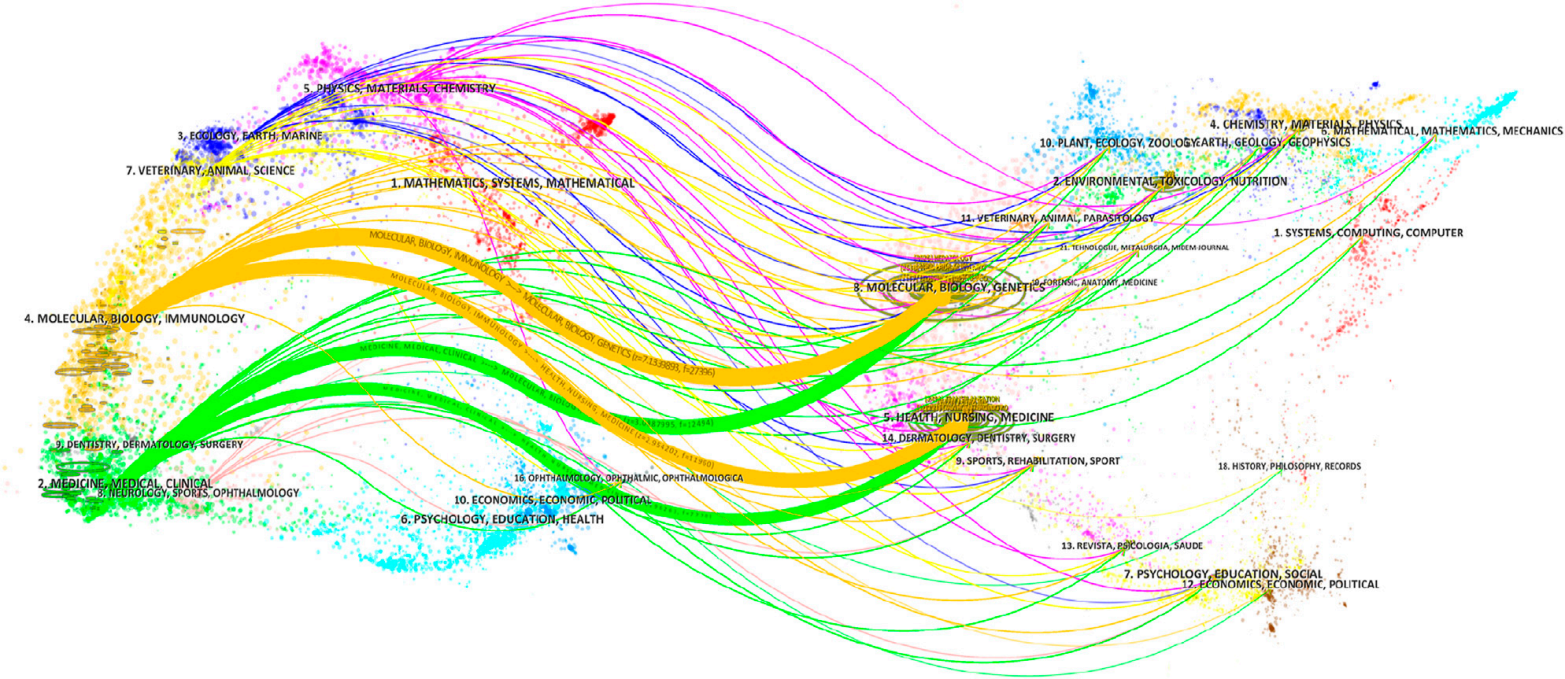
Journal publication volume and double figure overlay. The width of the line represents the strength of the connection.

**FIGURE 7:**
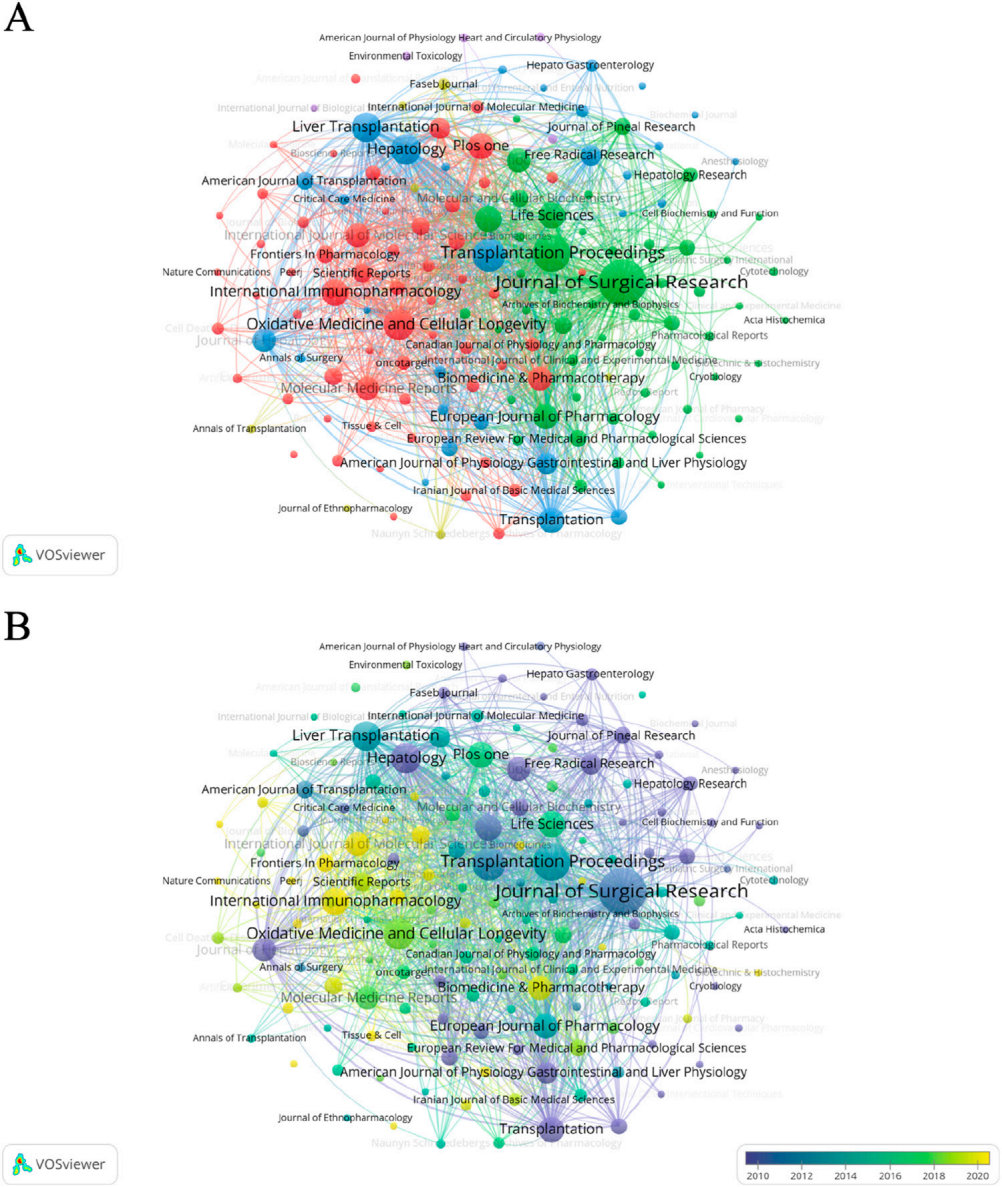
A graphical study of the network of connections between journals from 1995 to 2024. **(A)** Network of connections between journals on the topic of oxidative stress in hepatic ischemia-reperfusion injury. **(B)** Linkage network diagram of journals involved in the study of oxidative stress in hepatic ischemia-reperfusion injury. The size of the circle indicates the number of articles published in each journal, and the width of the connecting lines indicates the degree of collaboration between journals. Yellow color indicates recent publication in these journals, while purple nodes represent earlier publication in these journals.

Figure 6 shows the overlapping graphs of journal publication volume and subject areas. The publication areas most closely related to oxidative stress in the study of HIRI are molecular, biological, genetic, immunological, health, and clinical medicine. In addition, from the perspective of published journals, the Journal of Surgical Research has the largest number of publications, which is 84, followed by Transplantation Proceedings and Free Radical Biology and Medicine with 55 and 45 articles, respectively (Supplementary Table S3). Among them, as shown in Figure 7A, the classification can be mainly divided into surgery related, transplantation related and molecular immunology related fields. Figure 7B shows that in the early articles, the research was mainly conducted from the perspective of surgery and transplantation. In recent years, it has begun to focus on the basic research direction of combining molecular immunology.

### 3.4 Analysis of key authors

**FIGURE 8:**
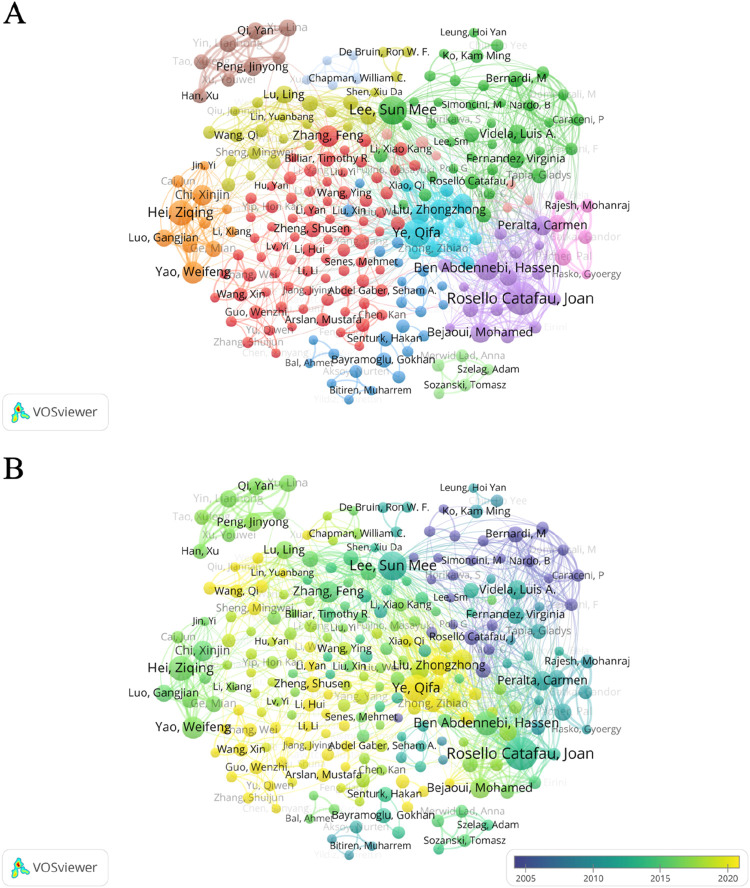
Collaborative network diagram study among authors from 1995 to 2024. **(A)** Collaborative network of authors on the topic of oxidative stress in hepatic ischemia-reperfusion injury. **(B)** Collaborative network diagram of authors in studies on oxidative stress in hepatic ischemia-reperfusion injury. The size of the circle indicates the number of articles published by each author, and the width of the connecting lines indicates the degree of collaboration between authors. Yellow color indicates articles published recently by the authors, while purple nodes represent articles published earlier by the authors.

Figure 8 shows the correlation of author information on the study of oxidative stress in HIRI. Rosello Catafau Joan, Lee Sun Mee, and Ye Qifa ranked first, second, and third with 28, 23, and 21 articles. Meanwhile, we sorted the authors by the number of publications from high to low and listed the citations of the top 15 authors (Supplementary Table S4). Figure 8A shows the collaboration between authors, among which Rosello Catafau Joan has the highest intensity of collaboration with other authors, followed by Ye Qifa and Ben Abdennebi Hassen. From the perspective of standardized citations of the authors, Mukhopadhyay Partha, Pacher Pal and Lee, Sun Mee are in the top three. Figure 8B shows the time of publication and the connection between the authors. Among the top three in terms of publication volume, Rosello Catafau Joan and Lee Sun Mee mainly published the most articles in the middle period, Ye Qifa published the most articles in the late period, and the earliest publications were Kumada K, Nakano H, and Yamaguchi M.

### 3.5 Keyword research and burst detection analysis

**FIGURE 9:**
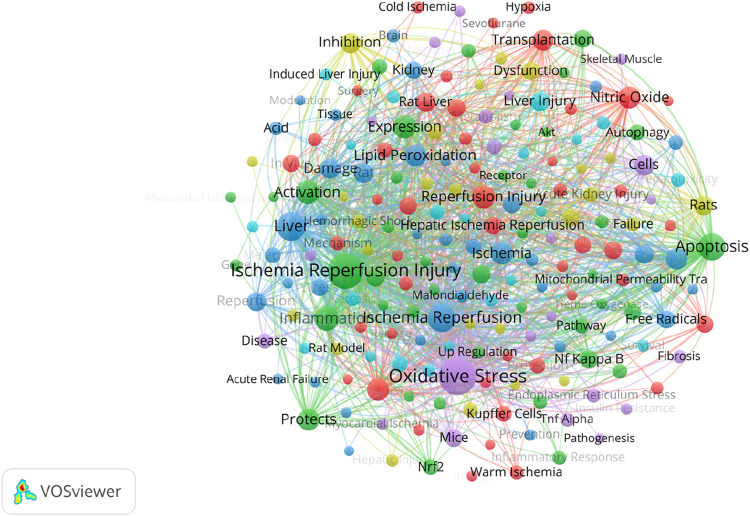
A study of keyword citation linkage network graphs from 1995 to 2024. The size of the circle indicates the number of citations for each keyword.

**FIGURE 10:**
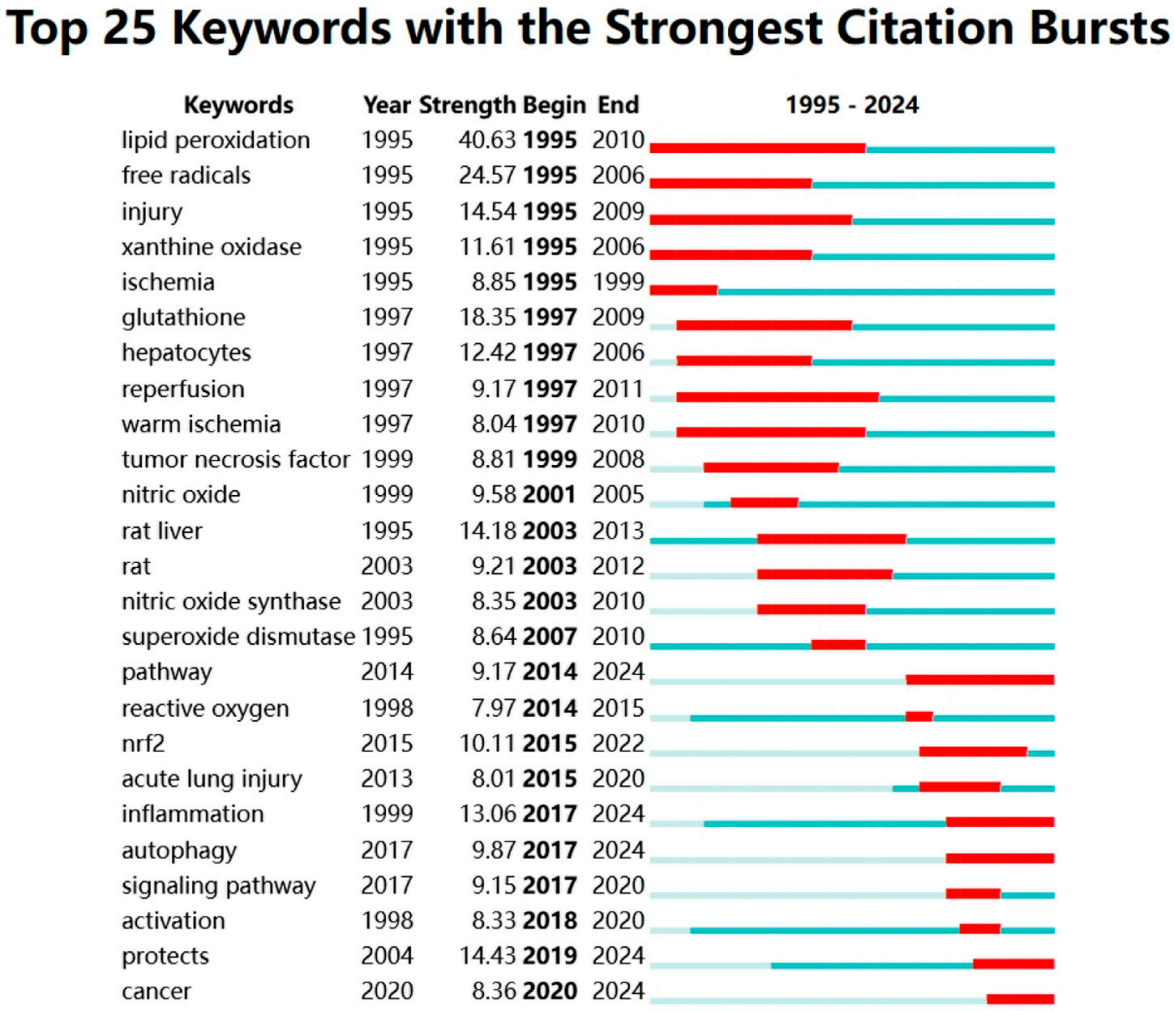
Keyword burst analysis. Year: time of the first appearance of the keyword; Intensity: the larger the value, the greater the intensity of the keyword appearance; Start: start time of the keyword burst; End: end time of the keyword burst. Red color represents the time of the keyword burst, dark green represents the time interval, and light green represents the keyword did not appear.

Figure 9 shows the high frequency keyword network diagram from 1995 to 2024. The keywords with the highest frequency are “Oxidative Stress,” “Ischemia Reperfusion Injury” and “Liver”. And it can be surprisingly found that the three keywords with the highest keyword association strength are consistent with the three keywords with the highest frequency (Supplementary Table S5). In addition, we can observe the keyword burst detection in the study of oxidative stress in HIRI, which is a method that can provide specific research areas and capture hot spots. The red line indicates that the keyword is frequently cited in each period. In contrast, the blue line indicates less popularity. First, the keyword “lipid peroxidation” appeared in 1995 with an importance index of 40.63. At the same time, “free radicals” (intensity 24.57) and “injury” (intensity 14.54) together formed the early outbreak keywords. After entering the mid-term, “rat liver” appeared in 2003 with an importance index of 14.18. At the same time, “rat” (intensity 9.21), “superoxide dismutase” (intensity 8.64) and “nitric oxide synthase” (intensity 8.35) together formed the mid-term burst keywords. Entering the late phase, “protects” appeared in 2019 with an importance index of 14.43. At the same time, “inflammation” (intensity 13.06) and “nrf2” (intensity 10.11) together formed the late burst keywords (Figure 10).

### 3.6 Analysis of research clusters

**FIGURE 11:**
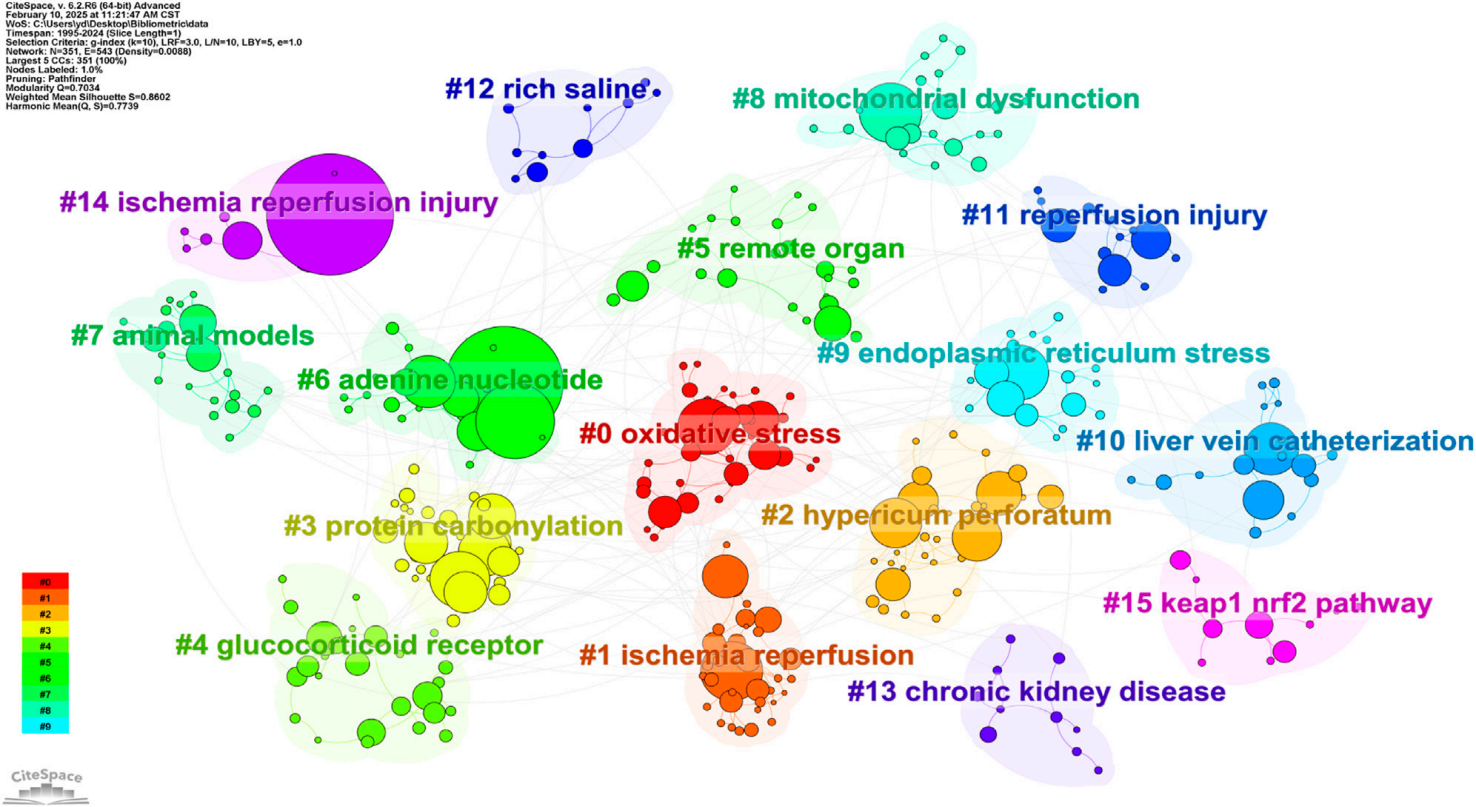
Cluster diagram of thematic research.

Figure 11 shows the research cluster analysis, which can summarize the popular topics and research directions in this field. The analysis results show that there are a total of 16 clusters, each with different keywords, reflecting the multifaceted nature of oxidative stress and its effects in HIRI. From cluster 0 to cluster 15, the keywords in these clusters decrease in order, and the topics of these clusters include “oxidative stress,” “ischemia reperfusion,” and “*hypericum perforatum*”.

## 4 Discussion

### 4.1 Oxidative stress in HIRI: overall trend and correlation analysis

The study of oxidative stress in HIRI has been a global endeavor, and over the past 30 years, research in this field has continued to receive global attention and the number of publications has continued to increase. China and the United States have been more prominent in this research, and the United States has dominated the middle decade, while China has dominated the last decade. However, although the total number of articles published in China is more than twice that of the United States, the total number of citations in China is lower than that of the United States. This indicates that the U.S. articles are of higher quality and the research is more innovative. In the 1990s, the U.S. began to observe the problem of HIRI and explored drugs to treat HIRI by alleviating oxidative stress, for example, some research on glutathione (Fukuzawa et al., 1995; Koeppel et al., 1998). Similarly, early studies were conducted in some of the strongest research countries, with Italian researchers identifying oxidative stress in human liver transplantation and the protective effect of exogenous coenzyme Q on HIRI by reducing oxidative stress (Biasi et al., 1995; Genova et al., 1999). Japanese researchers identified changes in the antioxidant glutathione in HIRI, and another study found that cyanidin 3-O-beta-D-glucoside alleviates HIRI by reducing lipid peroxidation (Tsuda et al., 1999).

In contrast, in the past decade, China has linked the effects of oxidative stress in HIRI with traditional Chinese medicine and its potential therapeutic applications. For example, resveratrol and tanshinone extracted from traditional Chinese medicine are used in the treatment of HIRI for their antioxidant and anti-inflammatory properties (Li et al., 2017; Wang et al., 2023; Liu Q. et al., 2024). This is somewhat different from the philosophy of countries such as the United States and European countries (Elimadi et al., 1998; Schauer et al., 2004; Kireev et al., 2013; Mao et al., 2022), which focus on the development of new synthetic drugs and targeted therapies. In addition, we can see that we started late compared to major research countries such as the United States, Italy and Germany, which may be due to the full implementation of our system of voluntary organ donation by citizens in 2015, as well as the popularization of the policy of donation to citizens after their death (DCD), which has led to an increase in the number of liver transplants and made us focus more on the problem of complications associated with liver transplantation (Huang, 2017).

In addition, from the perspective of journals, in the early days, the types of article submissions were biased toward surgical and clinical, and then slowly transitioned to basic, and related disciplines, but also medicine and molecules, immunity, combined with each other, and carried out from the clinic to ask questions, discover the phenomenon, and ultimately use the basis to solve the problem. Interdisciplinary collaboration has also played a crucial role in advancing the field. For example, the combination of pharmacology and bioinformatics has enabled the identification of novel antioxidant targets using artificial intelligence (AI)-driven approaches. Computational models have been used to design drugs and predict their efficacy, as well as to optimize their molecular structure to enhance therapeutic potential (Vamathevan et al., 2019; Pang et al., 2024). Despite these advances, the challenge of translating basic research into clinical applications remains. Much basic and clinical experimental confirmation is still needed.

### 4.2 Oxidative stress in HIRI: reviewing and highlighting previous studies

HIRI is a complex pathological process characterized by overproduction of ROS during the reperfusion phase, leading to oxidative stress and subsequent cellular damage (George et al., 2024). As can be seen from the results, Rosello Catafau Joan was the most prolific researcher, and the direction of his research focused on the optimization of preservation fluids and related pharmacological interventions in liver transplantation, which systematically explored the central role of oxidative stress in HIRI (Zaouali et al., 2014; Sánchez-Ramos et al., 2017; Panisello-Roselló et al., 2018). Lee Sun Mee focuses on the protective effects of drugs in HIRI and related pathways (Kim and Lee, 2007; Park and Lee, 2008; Kim et al., 2014). Ye Qifa focuses on the study of mechanisms related to hypothermic oxidative perfusion (HOPE) to alleviate ischemia-reperfusion injury in liver transplantation by alleviating oxidative stress (Zhang et al., 2021; Xiao et al., 2022). The authors investigated the role of hypothermic oxidative perfusion (HOPE) in combination with the MyD88 inhibitor TJ-5 to attenuate ischemia-reperfusion injury in liver transplantation. The study showed that HOPE combined with TJ-5 was able to significantly reduce HIRI by inhibiting oxidative stress, inflammation and the TLR/MYD88 signaling pathway, thereby improving the outcome of liver transplantation (Zhou et al., 2022). Among them, Lee Sun Mee’s research content has the highest total citations, which may indicate that the content of this researcher’s study has received more attention from subsequent researchers compared to the other two researchers.

Based on the analysis of keyword bursts, in which “lipid peroxidation” was the main keyword at the early stage, Moto Fukai investigated the progression of lipid peroxidation during hepatic ischemia and its effect on oxidative damage after reperfusion. The results showed that oxidative stress and lipid peroxidation during ischemia exacerbated oxidative damage after reperfusion, and the generation of lipid peroxidation products was positively correlated with the duration of ischemia (Fukai et al., 2005). In the medium term, the keyword “rat liver” dominates, and this is the time when researchers generally begin to use experimental animals to further investigate the basis of the relationship between oxidative stress and HIRI (Gedik et al., 2008). At a later stage, the keyword “protects” is dominant, whether it is the protection of the donor in liver transplantation or the protection of the liver in the postoperative period, which is the focus of research studies (Liu et al., 2019; Xiao et al., 2022), it is anticipated that future research will focus on investigating the protective effects in conjunction with mechanistic studies.

### 4.3 Emerging frontiers: nanotechnology and epigenetic modifications to mitigate oxidative stress

The field of mitigating oxidative stress in HIRI is rapidly evolving, and the focus of current research is on protective effects, including effective clinical treatment of HIRI. Nanotechnology and epigenetics are emerging as transformative frontiers. These approaches offer innovative solutions to the challenges of targeted therapy and precise regulation of redox homeostasis.

Nanotechnology enables targeted and controlled release of antioxidants, improving efficacy while minimizing off-target effects. Nanoparticles are a substance consisting of metal oxides or metal-related particles that can be used in antimicrobial, antioxidant and anticancer drug delivery applications (Padmanaban et al., 2023). Cerium oxide and manganese oxide nanocomplexes (CM NCs) were prepared and applied in the treatment of HIRI, and the results showed that CM NCs alleviated oxidative stress through the synergistic effects of scavenging ROS and oxygen (O_2_) generation, inhibited the activation of Kupffer cells and neutrophils, and reduced the secretion of inflammatory factors, demonstrating their clinical potential in the treatment of HIRI (Zhang S. et al., 2022).

Epigenetic modifications are heritable changes in cellular phenotype that primarily involve DNA methylation, histone modifications, and non-coding RNAs and are not affected by changes in DNA sequence (Zhang J. et al., 2022). Research on this modification is rapidly evolving. Recent articles include studies related to ischemia-reperfusion injury or transplantation and epigenetic modifications in other organs, including the kidney and heart (Xiang et al., 2022; Shi et al., 2023). In addition, epigenetic modifications that regulate oxidative stress and thereby attenuate HIRI have been reported in recent studies (Li Z. et al., 2024). Although epigenetic modification pathways in oxidative stress in HIRI have not been well studied, this may become one of the relevant therapeutic options in the future.

Despite this progress, significant challenges remain. One of the most pressing issues is the need for spatiotemporal regulation of redox homeostasis. Oxidative stress is a highly dynamic process, and its effects vary depending on the timing, location, and intensity of ROS production (Ji and Yeo, 2021). Precise control of these parameters will require the development of more sophisticated delivery systems and epigenetic modification modulators capable of responding in real time to changing redox environments.

Another major challenge is translating these findings from animal models to the clinic. Although preliminary studies have shown efficacy at the cellular or animal level, their safety and efficacy in humans have yet to be fully established. Nanotechnology and epigenetic modifications represent a new frontier in attenuating oxidative stress in HIRI with great potential for improving therapeutic outcomes. However, overcoming the challenges of spatiotemporal modulation and clinical translation will require continued innovation and interdisciplinary collaboration. Meanwhile, this study has some limitations. First, although the WoSCC database is the most well-known database for performing bibliometric analysis, there is a possibility that the literature may be somewhat incomplete due to inclusion issues. Secondly, because newer relevant literature may have been published during our writing process, it may not have been fully included in our study. However, by including enough articles, we believe that the potential impact of such issues is minimized.

## 5 Conclusion

Using bibliometric tools, we summarized and analyzed oxidative stress in HIRI studies from 1995 to 2024. The results show that China and the United States have made the largest contributions to the field so far. However, China is inferior to the United States in terms of the quality of relevant literature and should make more efforts in research innovation. Current research has gradually shifted from mechanistic studies to the study of related protective effects, which is more integrated with clinical treatments. In the therapeutic field, nanotechnology and epigenetic modifications may be the next promising therapeutic option. In the discussion, we analyze the development of this field in the last 30 years and provide some information for its future research.

## Data Availability

The raw data supporting the conclusions of this article will be made available by the authors, without undue reservation.
