# Supplementary material for: Knowledge graph and emerging trends in oxidative stress research on hepatic ischemia-reperfusion injury: a bibliometric analysis (1995–2024)
Source: Front Pharmacol. 2025 Jun 18;16:1587591. doi: 10.3389/fphar.2025.1587591 (PMC12215359; doi:10.3389/fphar.2025.1587591)
Supplement: Supplementary file 1 [file Table1.docx]

# Supplementary Table Appendix

**Supplementary Table 1**

**Top 15 high-output countries/regions**

| **Ranking** | **Country** | **Documents** | **Citations** |
| --- | --- | --- | --- |
| **1** | **CHINA** | **829** | **20985** |
| **2** | **USA** | **374** | **23438** |
| **3** | **TURKEY** | **243** | **4437** |
| **4** | **JAPAN** | **165** | **6268** |
| **5** | **GERMANY** | **105** | **3581** |
| **6** | **EGYPT** | **103** | **2070** |
| **7** | **SOUTH KOREA** | **97** | **3203** |
| **8** | **ITALY** | **88** | **3778** |
| **9** | **SPAIN** | **85** | **3206** |
| **10** | **FRANCE** | **64** | **1786** |
| **11** | **CANADA** | **61** | **2826** |
| **12** | **IRAN** | **61** | **1321** |
| **13** | **BRAZIL** | **49** | **1123** |
| **14** | **ENGLAND** | **47** | **3183** |
| **15** | **SAUDI ARABIA** | **42** | **1788** |

**Supplementary Table** 2

Top 15 high-output institutions

| **Ranking** | **Organization** | **Documents** | **Citations** |
| --- | --- | --- | --- |
| **1** | **Wuhan Univ** | **45** | **667** |
| **2** | **Sun Yat Sen Univ** | **42** | **1280** |
| **3** | **Shanghai Jiao Tong Univ** | **41** | **1727** |
| **4** | **Nanjing Med Univ** | **39** | **1122** |
| **5** | **Zhejiang Univ** | **38** | **1139** |
| **6** | **Dalian Med Univ** | **33** | **1595** |
| **7** | **Huazhong Univ Sci & Technol** | **31** | **832** |
| **8** | **Cent South Univ** | **30** | **493** |
| **9** | **Xi An Jiao Tong Univ** | **30** | **597** |
| **10** | **Univ Barcelona** | **29** | **1034** |
| **11** | **Univ Pittsburgh** | **28** | **2396** |
| **12** | **Inonu Univ** | **27** | **626** |
| **13** | **Sungkyunkwan Univ** | **27** | **1286** |
| **14** | **Csic** | **26** | **991** |
| **15** | **Gazi Univ** | **26** | **342** |

**Supplementary Table 3**

**Top 15 high-output** **journals**

| **Ranking** | **Source** | **Documents** | **Citations** |
| --- | --- | --- | --- |
| **1** | **Journal of Surgical Research** | **84** | **1920** |
| **2** | **Transplantation Proceedings** | **55** | **802** |
| **3** | **Free Radical Biology and Medicine** | **45** | **2660** |
| **4** | **Oxidative Medicine and Cellular Longevity** | **41** | **1173** |
| **5** | **Hepatology** | **39** | **2933** |
| **6** | **Liver Transplantation** | **38** | **1196** |
| **7** | **International Immunopharmacology** | **34** | **683** |
| **8** | **Life Sciences** | **31** | **702** |
| **9** | **Plos one** | **31** | **843** |
| **10** | **World Journal of Gastroenterology** | **31** | **1372** |
| **11** | **Transplantation** | **30** | **1101** |
| **12** | **European Journal of Pharmacology** | **29** | **937** |
| **13** | **Biomedicine & Pharmacotherapy** | **28** | **669** |
| **14** | **International Journal of Molecular Sciences** | **27** | **339** |
| **15** | **Shock** | **27** | **821** |

**Supplementary Table 4**

**Top 15 high-output** **authors**

| **Ranking** | **Authors** | **Documents** | **Citations** |
| --- | --- | --- | --- |
| **1** | **Rosello Catafau, Joan** | **28** | **767** |
| **2** | **Lee, Sun Mee** | **23** | **1202** |
| **3** | **Ye, Qifa** | **21** | **259** |
| **4** | **Hei, Ziqing** | **19** | **656** |
| **5** | **Ben Abdennebi, Hassen** | **18** | **402** |
| **6** | **Yao, Weifeng** | **16** | **608** |
| **7** | **Zhang, Feng** | **15** | **505** |
| **8** | **Bejaoui, Mohamed** | **14** | **212** |
| **9** | **Chi, Xinjin** | **14** | **442** |
| **10** | **Liu, Zhongzhong** | **14** | **183** |
| **11** | **Peralta, Carmen** | **13** | **488** |
| **12** | **Videla, Luis A.** | **13** | **324** |
| **13** | **Wang, Yanfeng** | **13** | **191** |
| **14** | **Lu, Ling** | **12** | **413** |
| **15** | **Peng, Jinyong** | **12** | **900** |

**Supplementary Table 5**

**Top 15 Keyword Occurrences and Total link strength**

| **Ranking** | **Keywords** | **Total link strength** | **Occurrences** |
| --- | --- | --- | --- |
| **1** | **Oxidative Stress** | **9278** | **1416** |
| **2** | **Ischemia Reperfusion Injury** | **7725** | **1211** |
| **3** | **Liver** | **4247** | **601** |
| **4** | **Ischemia Reperfusion** | **3776** | **544** |
| **5** | **Apoptosis** | **3483** | **486** |
| **6** | **Inflammation** | **2464** | **348** |
| **7** | **Activation** | **2142** | **306** |
| **8** | **Expression** | **2079** | **304** |
| **9** | **Reperfusion Injury** | **1951** | **290** |
| **10** | **Mechanisms** | **1829** | **258** |
| **11** | **Nitric Oxide** | **1783** | **258** |
| **12** | **Ischemia** | **1796** | **256** |
| **13** | **Lipid Peroxidation** | **1713** | **241** |
| **14** | **Protects** | **1698** | **239** |
| **15** | **Injury** | **1395** | **222** |
